# Effects of distribution of bone cement on clinical efficacy and secondary fracture after percutaneous kyphoplasty for osteoporotic vertebral compression fractures

**DOI:** 10.3389/fsurg.2022.1054995

**Published:** 2023-01-06

**Authors:** Zejun Pan, Quan Zhou, Ming Yang, Lei Deng, Xiayu Hu, Nanning Lv, Shaofeng Yang, Huilin Yang

**Affiliations:** ^1^ Department of Orthopaedics, The First Affiliated Hospital of Soochow University, Suzhou, China; ^2^ Department of Orthopedic Surgery, The Second People's Hospital of Lianyungang, Lianyungang, China

**Keywords:** PKP, bone cement, clinical efficacy, secondary vertebral fracture, OVCF

## Abstract

**Objective:**

To investigate the effect of bilateral bone cement distribution on the clinical efficacy of percutaneous kyphoplasty (PKP) in the treatment of osteoporotic vertebral compression fracture (OVCF).

**Methods:**

According to strict inclusion and exclusion criteria, 109 cases of OVCF patients treated with bipedicular PKP were included in this study from August 2018 to July 2020. According to the distribution morphology of bilateral bone cement in vertebral body, patients were divided into 3 groups, including Group A (*n* = 44): bilateral diffuse type; Group B (*n* = 31): bilateral dense type; Group C (*n* = 34): mixed type. To assess the clinical and radiographic efficacy of the surgery, the visual analogue scale (VAS) score, Oswestry disability index (ODI) score, anterior vertebral height (AVH), anterior vertebral height ratio (AVHR) and local kyphotic angle (LKA) were recorded at preoperatively, 2 days after surgery and 1 year after surgery.

**Results:**

Compared with the preoperative recorded value, the VAS score, ODI score, AVH, AVHR and LKA of the three groups were significantly improved at 2 days after surgery and 1 year after surgery (*p *< 0.05). At 1 year after surgery, the VAS score of Group A was better than that of groups B and C (*p *< 0.05), and there were significantly differences in ODI score, AVH, and LKA between Group A and Group B (*p *< 0.05). Compared with other bone cement distribution patterns, the incidence of recompression in bilateral diffuse bone cement distribution pattern was lower (*p *< 0.05).

**Conclusion:**

In the mid-term follow-up of patients undergoing bipedicular PKP, diffuse and symmetrical distribution of bone cement can obtain better clinical improvement and lower the incidence of secondary compression.

## Introduction

Osteoporosis is a systemic disease with bone mass reduction and bone tissue microarchitectural changes, which leads to increased bone fragility and is prone to fracture of hip, wrist and vertebral body (1). It is estimated that osteoporosis affects about 200 million women worldwide, with 8.9 million cases of osteoporosis fractures occurring every year (2, 3). Although conservative treatment such as braking and taking analgesic drugs can alleviate acute pain, complications such as bedsore, pneumonia and bone loss caused by bed rest will further affect the prognosis of patients and bring huge social and economic burden (4–6).

Vertebral augmentation (VA) has been widely used in the past decades and has become an important surgical treatment for osteoporotic vertebral compression fracture (OVCF). As an improved technique of percutaneous vertebroplasty (PVP), PKP has been proved to be an effective measure for the treatment of OVCF. In addition to fixing fracture blocks, bone cement also relieves pain by destroying local nerves through thermochemical toxicity (7, 8). PKP can restore the height of vertebral body, reduce the kyphosis angle of the compressed vertebral body and restore the physiological lordosis of the lumbar spine, so as to reconstruct the sagittal balance of patients' spine (9). In addition, PKP can reduce the pressure during bone cement infusion and increasing the amount of bone cement infusion while decrease the risk of bone cement leakage (10).

As a common complication of VA, secondary compression is increasing with more and more patients undergoing VA. The reported adjacent vertebral fractures (AVFs) of VP (8%–52%) and KP (3%–29%) vary widely (11–13). The distribution of bone cement is considered to be closely related to the relief of postoperative pain, recompression of the operative vertebral body and new compression of the adjacent vertebral bodies (14). In the study of Tanigawa et al., compact and solid cement filling pattern was more prone to fracture at adjacent segments than sponge like cement filling pattern (15). Compared with unipedicular PKP, bone cement of bipedicular PKP can be better distributed on both sides of the vertebral body, so as to create a more uniform biomechanical balance (16). However, no study has been found to comprehensively analyze the influence of the distribution and the symmetry of bilateral bone cement.

In this study, we retrospectively studied the effects of different bone cement distribution patterns on radiographic indices, clinical efficacy and secondary compression in OVCF patients after PKP treatment.

## Method

### Clinical cases

The inclusion criteria were as follows: (1) Single segment compression fracture of thoracic or lumbar spine. (2) The vertebra was hyperintense on T2-weighted images, hypointense on T1-weighted images and hyperintensity on short time inversion recovery (STIR). (3) The average bone mineral density (BMD) of lumbar vertebra was less than −2.5 on Dual-energy *x*-ray absorptiometry (DEXA). (4) No previous history of VA surgery. (5) At least 1 year of follow-up.

The exclusion criteria were as follows: (1) Other pathological fracture of vertebral body, such as myeloma, metastatic cancer, hemangioma, tuberculosis. (2) Patients who combined with damage of spinal cord or nerve root. (3) Patients need to use hormones for a long time.

**Figure 1:**
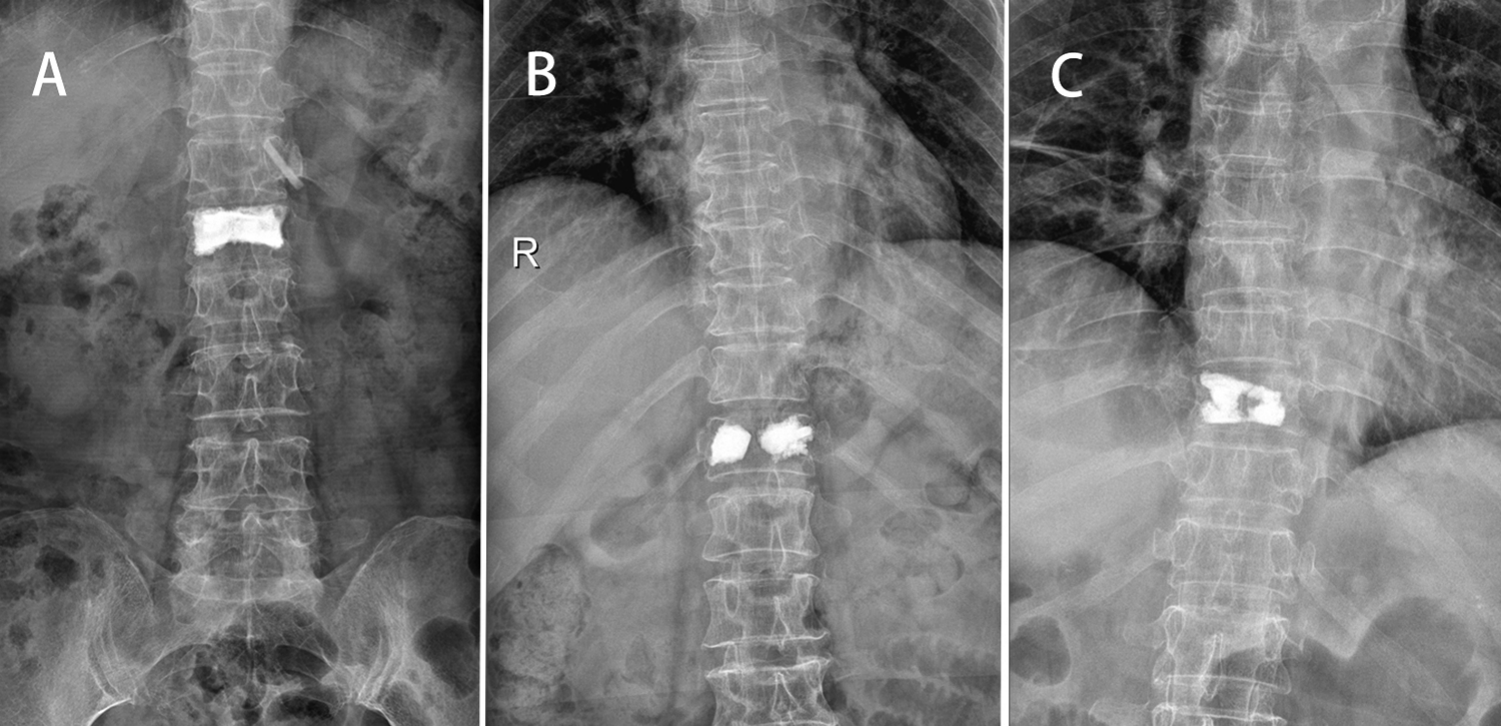
In Group A, bilateral diffuse bone cement can be seen on the postoperative anteroposterior radiograph (**A**). In Group B, bilateral dense cement can be seen on the postoperative anteroposterior radiograph (**B**). In Group C, both diffuse and dense bone cement can be seen on the postoperative anteroposterior radiograph (**C**).

According to strict inclusion and exclusion criteria, a total of 109 patients were performed PKP for painful OVCF in our hospital from August 2018 to July 2020. All patients underwent *x*-ray, CT and magnetic resonance before operation to determine the fracture vertebral body. And the BMD of lumbar spine (L1–L5) was measured by DEXA. The bone cement dispersion on both sides of the vertebral body was scored according to the bone cement distribution pattern observed on frontal radiographs. (Dispersion scoring standard: 2 points for bone cement dispersion to the upper endplate or the lower endplate respectively and 1 point for bone cement dispersion to the middle line or the outer edge respectively. If the score is ≥5, it is considered as diffuse type, otherwise it is dense type) Group A: bilateral diffuse type (Figure 1A); Group B (Figure 1B): bilateral dense type; Group C: mixed type (Figure 1C).

### Surgical techniques

After general anesthesia, patients lied prone on the bed to obtain a preliminary reposition of the vertebral body. Then, the C-arm fluoroscopy and the patients' position were adjusted to obtain standard anteroposterior and lateral images (the bilateral pedicle on the anteroposterior image was symmetrical and the pedicle on the lateral image was overlapped). The puncture point was located on the outer upper side of the bilateral pedicle (in the direction of 2 o'clock and 10 o'clock). During the puncture process, the puncture direction was constantly adjusted according to the perspective picture, gradually reaching about 3 mm in front of the posterior edge of the vertebral bodies. Then the guide wires, the expansion cannulas and the working cannulas were performed in turn, and Inflatable balloons were used to further restore the height of the vertebral body. Finally, polymethylmethacrylate (PMMA) cement was carefully pushed under radiation monitoring until the anterior two-thirds of the vertebral body were well filled. The patients were allowed to walk 12 h after surgery. All patients were treated with anti-osteoporotic treatment after surgery.

### Assessed parameters

The basic information of each patient, including age, gender, bone mineral density value and surgical segment was recorded. The vertebral fractures in each group were classified and compared according to Magerl type (17). The volume of bone cement, operation duration and blood loss were recorded. The visual analogue scale (VAS) score from 0 (no pain) to 10 (maximal imaginable pain) was used to assess the subjective pain feeling of patients at preoperatively, 2 days after surgery and 1 year after surgery. At the same time, the Oswestry disability index (ODI) score was used to evaluate the improvement of patients' life.

In terms of imaging, the anterior vertebral height (AVH), anterior vertebral height ratio (AVHR) and local kyphosis angle (LKA) were measured and analyzed on standing lateral plain radiography preoperatively, 2 days after surgery and 1 year after surgery to evaluate the vertebral reduction. The AVH was defined as the anterior height of the fractured vertebral body. The AVHR refers to the ratio of the anterior height of the fractured vertebral body to the average anterior height of the upper and lower adjacent vertebral bodies. The LKA is the kyphosis Cobb angle of the fracture vertebral body, which is the angle between the upper endplate and the lower endplate of the fractured vertebra.

### Statistical analysis

The statistical analysis was performed with SPSS 26.0 software (SPSS Inc., Chicago, IL) and the results were presented as the mean and SD. *χ*^2^ test was used to analyze the differences of categorical variables among the three groups. ANOVA test was used to analyze the differences of continuous variables among the three groups. The paired *t*-test was used to compare the difference between the postoperative and preoperative indexes within-group. *p *< 0.05 was considered to indicate statistical significance.

## Results

### Demographic data

A total of 87 women and 22 men (50 thoracic vertebras and 59 lumber vertebras) underwent PKP treatment. The mean age was 71.27 ± 6.77 years. There was no significant difference in age, sex, fracture segment, Magerl type and BMD among the three groups (*p *> 0.05). The mean operation time of each group was 39.89 ± 7.08 min, 38.94 ± 8.74 min and 40.82 ± 7.06 min, respectively. The mean blood loss of each group was 14.07 ± 3.36 ml, 13.55 ± 3.60 ml and 13.24 ± 3.10 ml, respectively. The injection volume of bone cement in Group A was higher than that in Group B and the difference was statistically significant (*p *= 0.008) (Table 1).

**Table 1:** General characteristics of the patients.

Characteristic	Group A	Group B	Group C	*p*-Value
Number of patients	44	31	34	–
Age (years)	71.64 ± 5.40	70.61 ± 7.06	71.38 ± 8.21	0.811
Gender (F/M)	31/9	24/6	29/7	0.991
BMD (T-score)	3.26 ± 0.48	3.30 ± 0.43	3.23 ± 0.46	0.861
Magerl type				0.824
A1.1	14	7	9	
A1.2	18	12	15	
A1.3	12	12	10	
Surgical segment				0.569
Thoracic	18	14	18	
Lumbar	26	17	16	
Operation duration (mins)	39.89 ± 7.08	38.94 ± 8.74	40.82 ± 7.06	0.606
Volume of bone cement	8.23 ± 1.54	7.39 ± 1.28^b^	7.91 ± 1.06	**0**.**030**^a^
Blood loss (ml)	14.07 ± 3.36	13.55 ± 3.60	13.24 ± 3.10	0.542

M, male; F, female; BMD, bone mineral density.

^a^ By ANOVA test.

^b^ Compared with Group A, *p *< 0.05.

### Radiologic parameters

At 2 days and 1 year after operation, AVH and AVHR of all groups were significantly improved compared with those before surgery (*p *< 0.05). Similarly, Cobb angle was significantly improved after surgery (*p *< 0.05). These Radiologic parameters had no statistical difference between 2 days and 1 year after surgery (*p *> 0.05). There was no significant difference in AVH, AVHR and LKA among the three groups at preoperative and 2 days after surgery (*p *> 0.05). At 1 year after operation, AVH and LKA were statistically different between Group A and Group B (*p *< 0.05, Table 2).

**Table 2:** Comparison of radiographic parameters.

	Group A	Group B	Group C	*p*-Value
AVH				
Preop	19.39 ± 4,29	18.92 ± 4.36	19.77 ± 4.06	0.721
Postop 2d	23.59 ± 3.69^a^	22.77 ± 3.78^a^	23.24 ± 3.74^a^	0.646
Postop 1y	23.39 ± 3.65^a^	21.65 ± 3.57^ab^	22.57 ± 3.68^a^	0.128
AVHR (%)				
Preop	68.75 ± 15.22	66.02 ± 14.00	69.85 ± 14.22	0.555
Postop 2d	82.68 ± 13.10^a^	79.60 ± 12.19^#^	82.10 ± 13.00^a^	0.574
Postop 1y	80.98 ± 12.93^a^	76.63 ± 11.03^#^	80.62 ± 12.68^a^	0.280
LKA (°)				
Preop	16.38 ± 5.54	17.60 ± 5.97	16.81 ± 5.43	0.656
Postop 2d	9.03 ± 4.16^a^	9.90 ± 4.04^a^	9.87 ± 4.11^a^	0.573
Postop 1y	9.57 ± 4.33^a^	11.60 ± 3.78^ab^	10.45 ± 4.45^a^	0.128

AVH, anterior vertebral height; AVHR, anterior vertebral height ratio; LKA, local kyphotic angle.

^a^ Compared to pre-operation, *p *< 0.05.

^b^ Compared with Group A, *p *< 0.05.

### Clinical outcomes

**Figure 2:**
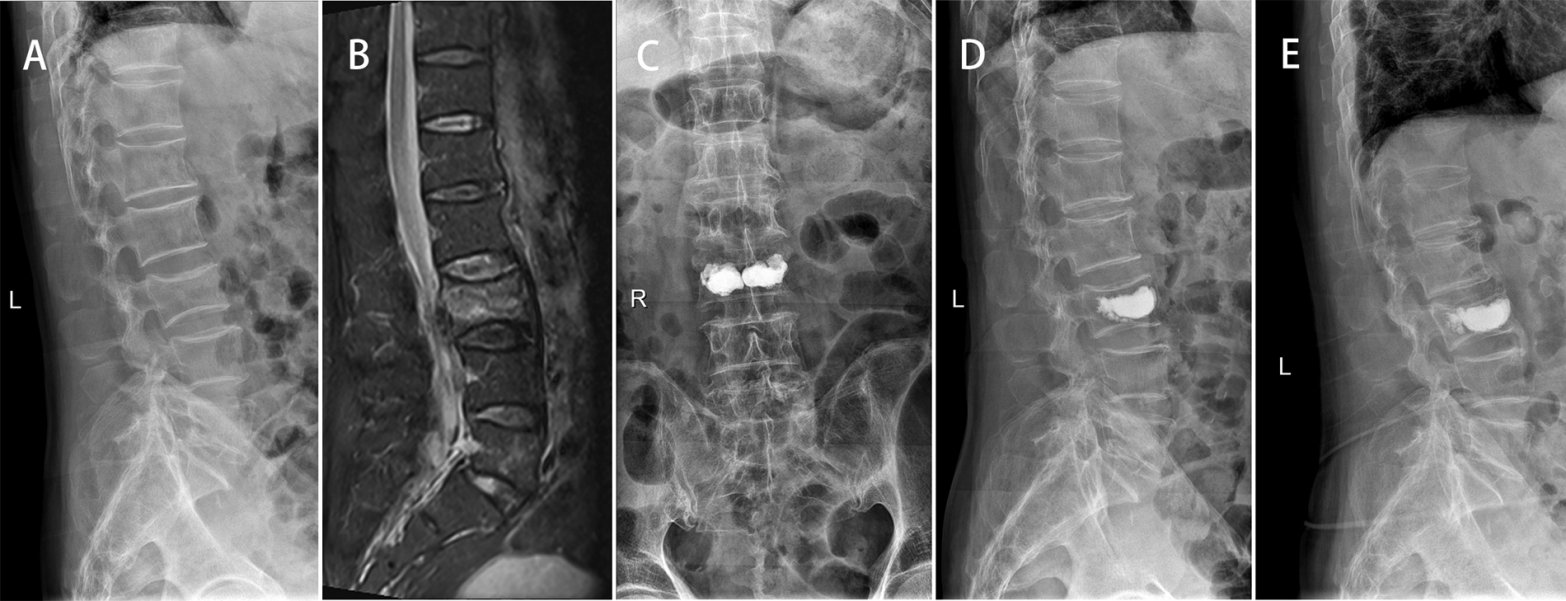
A 75-year-old male patient with L3 OVCF developed painless recompression of the operative vertebra. *X*-ray showed a wedge-shaped change of L3 (**A**). MR showed a fresh compression fracture of L3 (**B**). After PKP surgery, *x*-ray showed that the cement was densely distributed on both sides of the vertebra (**C**) and the height of L3 vertebral body was improved compared with that before operation (**D**). At 1-year follow-up, *x*-ray showed that L3 vertebral body recompressed and kyphosis was aggravated (**E**).

**Figure 3:**
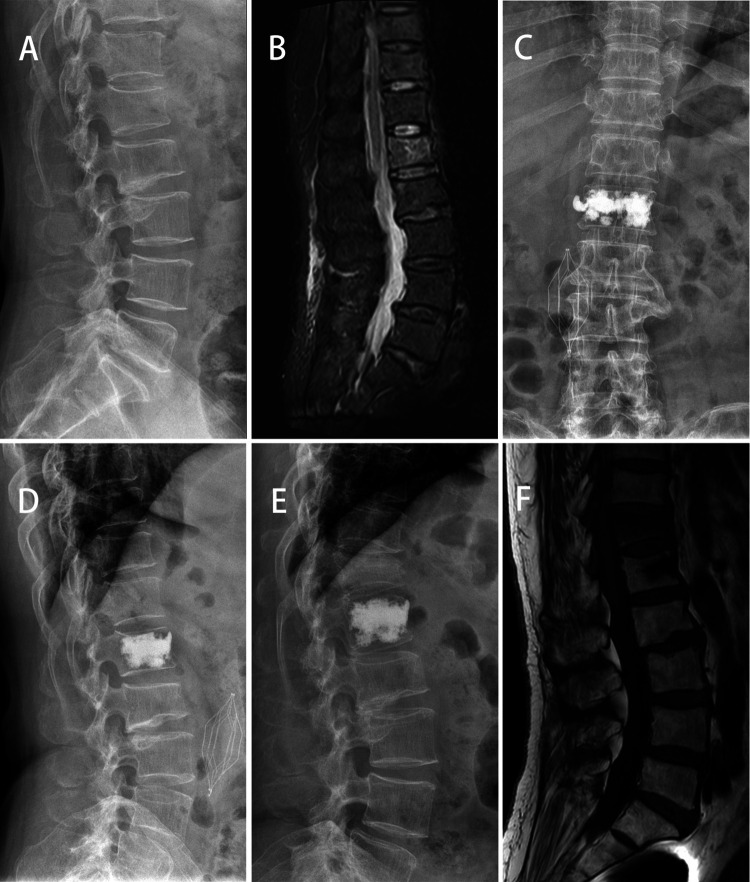
A 64-year-old female patient with L1 vertebral compression fractures developed adjacent vertebral compression. *X*-ray showed and MR showed a fresh compression fracture of L1(**A,B**). After PKP surgery, *x*-ray showed that the bone cement on one side of the vertebra was densely distributed (**C,D**). At 12 months after surgery, the patient developed T12 OVCF (**E,F**).

The VAS score and ODI score of the three groups were significantly improved at 2 days and 1 year after surgery (*p *< 0.05). At 1 year after operation, the VAS score of Group A was lower than that of Group B and C (*p *< 0.05). There was a significant difference in ODI score between Group A and Group B at 1 year after operation (*p *< 0.05) (Table 3). At 1 year after operation, the secondary vertebral fracture incidence of in Group A was lowest (6.8%, 3 cases of adjacent vertebra), followed by 29.0% in Group B (3 cases of adjacent vertebra and 6 cases of cemented vertebra) and 23.5% in Group C (4 cases of adjacent vertebra and 3 cases of cemented vertebra) (*p *< 0.05) (Figures 2, 3).

**Table 3:** Comparison of functional outcomes.

	Group A	Group B	Group C	*p*-Value
VAS score				
Preop	7.59 ± 0.87	7.81 ± 0.91	7.44 ± 1.05	0.296
Postop 2d	2.36 ± 1.04^c^	2.55 ± 0.81^c^	2.68 ± 1.04^c^	0.368
Postop 1y	1.66 ± 0.89^c^	2.20 ± 0.79^cd^	2.09 ± 0.79^cd^	**0**.**014**^a^
ODI				
Preop	59.84 ± 8.99	58.55 ± 9.84	58.82 ± 8.55	0.806
Postop 2d	24.61 ± 3.19^c^	25.13 ± 2.68^c^	24.68 ± 2.96^c^	0.740
Postop 1y	18.57 ± 4.55^c^	20.71 ± 4.52^cd^	19.82 ± 3.89^c^	0.106
Secondary fracture	3	9	8	**0**.**032**^b^
Cemented vertebrae	0	6	3	
Adjacent vertebrae	3	3	5	

VAS, Visual Analogue Scale; ODI, Oswestry Disability Index.

^a^ By ANOVA test.

^b^ By *χ*^2^ test.

^c^ Compared to pre-operation, *p *< 0.05.

^d^ Compared with Group A, *p *< 0.05.

## Discussion

VA is a widely used treatment for painful osteoporotic vertebral fracture, which avoids the risk of screw loosening and pulling out caused by internal fixation surgery in patients with osteoporosis, and alleviates the symptoms of patients through less trauma (18, 19). Previous studies have discussed the clinical effect of bone cement distribution in the vertebral body on VA (20). The distribution of bone cement in vertebral body is affected by many factors such as bone cement viscosity, bone density and puncture technique (21). Previous studies have shown that hematoma in the fracture space after fracture and fibrous repair tissue after 2 weeks will hinder the dispersion of bone cement (22). In addition, compared with low viscosity bone cement, high viscosity bone cement can disperse more evenly and reduce the risk of leakage.

Liu et al. showed that the confluent rather than separated bilateral bone cement pattern made patients get better postoperative clinical improvement (23). He et al. believed that the “H” type bone cement distribution had a larger contact area with the cancellous bone of the vertebral body, which can increase the combination between the bone cement and the bone trabecula, thus reducing the fretting between the two and relieving the residual pain (14). However, there are no studies that comprehensively consider the influence of bone cement diffusion to the upper and lower endplates, midline and lateral side on the prognosis of patients. In addition, the symmetry of bilateral bone cement is often neglected in previous studies. In this study, bone cement was divided into diffuse type and dense type according to the two-dimensional distribution of bone cement on the frontal radiographs. Meanwhile, the factor of bilateral bone cement symmetry was considered to analyze its impact on the clinical efficacy and secondary fracture. Obviously, the VAS score and ODI score of each group after PKP were significantly improved compared with those before PKP, which proves that PKP can effectively relieve the pain symptoms of patients and improve their postoperative living conditions. OVCF brings not only acute pain but also problems caused by secondary kyphosis. Different types of OVCF lead to different degrees of vertebral height loss. In Magerl type, patients of A1.2 and A1.3 had more AVH loss (17). Overall, the risk of secondary fracture is closely related to the degree of compression of the fractured vertebral body (24, 25). The increase of local kyphosis and the change of sagittal balance caused by the compression of OVCF anterior column will increase the load on the anterior column and ultimately promote the occurrence of AVF (24, 26). The AVH, AVHR and Cobb angles of OVCF patients after PKP were significantly improved compared with pre-operation. Therefore, it is pretty necessary for PKP to restore the anterior height of vertebral body, rectify kyphosis and maintain the sagittal balance of patients' spine while alleviating the pain of patients.

The re-loss of cement vertebral height was found in some patients underwent VA. The vertebral body height of some patients regressed to the preoperative state, and even underwent revision surgery again (27). Some studies have found that the recompression of cemented vertebrae after VA surgery may be related to a variety of factors, such as the degree of osteoporosis, osteonecrosis, the distribution of bone cement and the degree of kyphosis correction (28, 29). The lump distribution of bone cement is considered to be an important risk factor for cement vertebral body recompression after VA (21, 30). This distribution pattern of bone cement can't improve the strength of bone around the vertebral body, resulting in a high risk of secondary fracture. Kim et al. found that the loss of PVP vertebral body height was smaller than that of PKP in the study of cadaveric vertebral bodies (31). Compared with PVP, the distribution of bone cement in PKP is more inclined to form lump, which may be related to the barrier formed by surrounding bone compression during balloon expansion affecting the diffusion of bone cement around the vertebral body (28). Therefore, more attention should be paid to the influence of cement distribution pattern in PKP on the cement vertebral body recompression after surgery. Li et al. believed that too large the distance between PMMA and endplate (DBPE) would increase the incidence of cemented vertebral body recompression (32). This is consistent with our conclusion that patients with bilateral poor diffusion are more likely to occur cement vertebral body recompression. Moreover, we found that some patients in the bilateral dense group had varying degrees of vertebral height loss one year after surgery. Most of these patients did not have sudden low back pain symptoms during follow-up, but decreased vertebral body height and increased Cobb angle were found on follow-up imaging examinations. This may be caused by the destruction of peripheral nerve endings and bone due to the thermochemical toxicity of bone cement (8). These patients experienced multiple mild, painless subclinical fractures after surgery resulting in loss of vertebral height.

The symmetry of cement distribution in vertebral body has been concerned by many scholars. In vitro biomechanical research showed that the bone cement tended to be “H” shape distribution during bipedicular PKP, which can better maintain the bilateral biomechanical balance of the vertebral body (16). However, when unipedicular PKP was performed, more “O” shape cement distribution would increase the risk of recompression on the weak side (16, 33). Similarly, we observed that when the two sides of bone cement were distributed asymmetrically, the vertebral body augmented with cement was prone to recompression in bipedicular PKP. When the vertebral body is subjected to axial load, the bone on the side with poor dispersion of bone cement is more likely to recompress due to insufficient bone cement support. We found bone cement with asymmetric distribution is not only more likely to lead to cemented vertebral fracture, but also increases the risk of AVFs. The augmented effect of cement on the vertebral body will transfer the abnormal load to the adjacent vertebral body, leading to the accelerated failure of the adjacent vertebral body (34). We consider that when the bone cement is unevenly distributed on both sides of the vertebral body, the adjacent vertebral body contacting the diffuse side of the cemented vertebral body will bear more stress, which will lead to the AVFs.

### Limitation

This study still has some limitations. First, this study is a retrospective study. The number and time of follow-up patients are limited, which may affect the reliability of the conclusions. In addition, due to the limitations of two-dimensional images, there is still a certain difference between the actual dispersion of bone cement and the dispersion of bone cement judged only by positive *x*-ray. For more accurate evaluation of bone cement distribution, 3D CT will be a better choice. Moreover, this study indirectly reflects the support range of bone cement on the upper and lower endplates by the lateral distribution of bone cement on the frontal radiographs. However, bone cement diffusion to the edge and the middle line does not mean that the end plate contacts more widely at the horizontal level in a few cases.

## Conclusion

In general, the diffuse and symmetrical distribution of bone cement in bilateral pedicle PKP achieved better clinical improvement in the mid-term follow-up. At the same time, the bilateral diffuse group helps to reduce the occurrence of secondary compression during follow-up.

## Data Availability

The raw data supporting the conclusions of this article will be made available by the authors, without undue reservation.
